# Oxysterols in the brain of the cholesterol 24-hydroxylase knockout mouse

**DOI:** 10.1016/j.bbrc.2014.01.153

**Published:** 2014-04-11

**Authors:** Anna Meljon, Yuqin Wang, William J. Griffiths

**Affiliations:** Institute of Mass Spectrometry, College of Medicine, Swansea University, Singleton Park, Swansea SA2 8PP, UK

**Keywords:** 24S-Hydroxycholesterol, 24R-Hydroxycholesterol, 24S,25-Epoxycholesterol, Oxysterol, Brain, Cyp46a1, Cyp, cytochrome P450, ESI, electrospray ionisation, FT, Fourier transform, GP, Girard P reagent, INSIG, insulin induced gene, LC, liquid chromatography, Lxr, liver x receptor, MRM, multiple reaction monitoring, MS, mass spectrometry or mass spectrum, MS^n^, mass spectrometry with multistage fragmentation, RIC, reconstructed ion chromatogram, RP, reversed phase, SCAP, SREBP cleavage-activating protein, SPE, solid phase extraction, SREBP, sterol regulatory-element binding protein, TLR, Toll-like receptor, wt, wild type

## Abstract

Oxysterols are oxidised forms of cholesterol or its precursors. In this study we utilised the cholesterol 24-hydroxylase knockout mouse (*Cyp46a1*−/−) to study the sterol and oxysterol content of brain. Despite a great reduction in the abundance of 24S-hydroxycholesterol, the dominant metabolite of cholesterol in wild type brain, no other cholesterol metabolite was found to quantitatively replace this oxysterol in the *Cyp46a1*−/− mouse. Only minor amounts of other side-chain oxysterols including 22R-, 24R-, 25- and (25R),26-hydroxycholesterols were detected. In line with earlier studies, levels of cholesterol were similar in *Cyp46a1*−/− and wild type animals. However, the level of the cholesterol precursor, desomsterol, and its parallel metabolite formed via a shut of the mevalonate pathway, 24S,25-epoxycholesterol, were reduced in the *Cyp46a1*−/− mouse. The reduction in abundance of 24S,25-epoxycholesterol is interesting in light of a recent report indicating that this oxysterol promotes dopaminergic neurogenesis.

## Highlights

• 24S-Hydroxycholesterol is almost absent from brain of the *Cyp46a1*−/− mouse.
• It is not quantitatively replaced by another oxysterol.
• Minor amounts of 22R-, 24R-, 25- and (25R),26-hydroxycholesterols are present.
• Cholesterol biosynthesis is reduced in brain of the *Cyp46a1*−/− mouse.
• 24S,25-Epoxycholesterol synthesis is reduced in brain of the *Cyp46a1*−/− mouse.

## Graphical abstract

**Figure:**
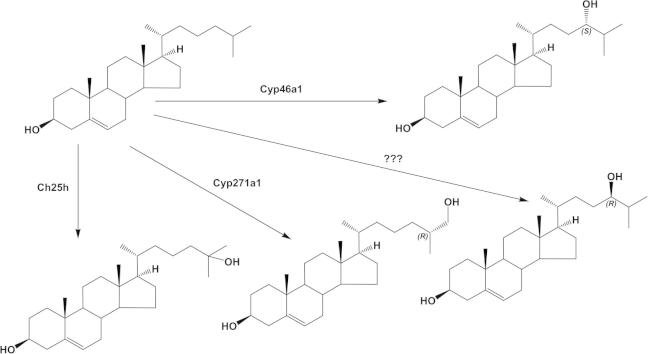

## 1 Introduction

Hydroxylation of cholesterol to give 24S-hydroxycholesterol, in a reaction catalysed by the enzyme cytochrome P450 (CYP) 46A1, represents the major route for removal of cholesterol from the brain of mouse and man [1,2]. 24S-Hydroxycholesterol can cross the blood brain barrier and is transported in the blood stream to the liver where it is metabolised to bile acids [3]. The mouse enzyme, Cyp46a1, is expressed in neurons in brain and at low levels in liver [4]. The human enzyme, CYP46A1, is expressed in neurons in normal human brain and also in glial cells in Alzheimer’s disease patients [5]. Earlier studies of the *Cyp46a1*−/− mouse revealed a reduction in *de novo* cholesterol synthesis in brain and indicated that brain cholesterol homeostasis is maintained via reduced biosynthesis rather than via up-regulation of an alternative metabolising enzyme [6]. The *Cyp46a1*−/− mouse shows impaired learning and memory, and further studies revealed that this is a consequence of a reduced flow of metabolites through the cholesterol biosynthetic pathway (also known as the mevalonate pathway) [7,8].

Oxysterols have been shown to be more than transport forms of cholesterol, and there is mounting evidence that they represent important signalling molecules. They may exert biological activity as ligands to INSIG (insulin induced gene) and thereby modulate cholesterol biosynthesis by retaining SCAP (SREBP cleavage-activating protein) and SREBP-2 (sterol regulatory-element binding protein 2) within the endoplasmic reticulum, thus attenuating the formation of mature SREBP-2, the master transcription factor of the cholesterol biosynthetic pathway [9,10]. Oxysterols also act as ligands to nuclear receptors. Those with a side-chain hydroxy-, oxo-, epoxy- or carboxylate group can act as ligands to the liver x receptors (Lxr) which are expressed in brain [11,12]. Recent reports indicate that activation of Lxr by oxysterols can induce neurogenesis of dopaminergic neurons [13,14]. In normal rodent brain the oxysterol profile is completely dominated by 24S-hydroxycholesterol [15,16], obscuring the possible presence of alternative cholesterol metabolites. The *Cyp46a1*−/− mouse thus provides an excellent opportunity to profile low-level oxysterols, with potential biological activity, without being obscured by the overwhelming background of 24S-hydroxycholesterol.

## 2 Methods

### 2.1 Reagents and standards

Sterol standards were obtained from Avanti Polar Lipids (Alabaster, Alabama, USA). Cholesterol oxidase from *Streptomyces* sp*.* was from Sigma–Aldrich (Dorset, UK). Girard P (GP) reagent [1-(carboxymethyl)pyridinium chloride hydrazide] was from TCI Europe (Oxford, UK). Reversed phase (RP) solid phase extraction (SPE) cartridges, Certified Sep-Pak C_18_, 200 mg, were from Waters Inc (Elstree, UK). Solvents and other reagents were from Fisher-Scientific (Loughborough, UK), VWR (Lutterworth, UK) and Sigma–Aldrich.

A stock solution of deuterated 24R/S-hydroxycholesterol standard was prepared by dissolving 1 mg of 24R/S-[26,26,26,27,27,27-^2^H_6_]hydroxycholesterol in propan-2-ol (10 mL). Ten μL of this stock solution was diluted with 990 μL of ethanol to make a working solution of 1 ng/μL. A solution of deuterated cholesterol was prepared by dissolving 10 mg of [25,26,26,26,27,27,27-^2^H_7_]cholesterol in 10 mL of propan-2-ol to make a solution of 1 μg/μL.

### 2.2 Extraction of sterols and oxysterols from brain

Brain extracts were a generous gift from David Russell, University of Texas Southwestern Medical Center at Dallas. Lipids were extracted from brains of four 15 week old female mice, two *Cyp46a1*−/− animals and two wild type (wt, *Cyp46a1+*/*+*) controls [6]. Brain tissue (0.4–0.5 g, wet weight) was homogenised in PBS (1.2 mL) and CHCl_3_:CH_3_OH (1:2, v/v, 6 mL) added. After further homogenisation the mixture was centrifuged (1360*g*) for 10 min at 4 °C. The supernatant was decanted to a fresh tube and CHCl_3_ (2 mL) and PBS (2 mL) were added. Following further centrifugation the organic phase was removed and solvents evaporated [17]. Lipid extracts each corresponding to 100 mg of brain wet weight were dissolved in 1.05 mL of ethanol containing 50 ng of 24R/S-[^2^H_6_]hydroxycholesterol and 50 μg of [^2^H_7_]cholesterol and ultrasonicated for 15 min at room temperature. To the mixture 0.45 mL of water was added and the extract was sonicated for another 15 min. The mixture was centrifuged (14,000*g*, 4 °C, 60 min) and the supernatant was collected. The residue was extracted a second time and the supernatants combined.

Next, oxysterols were separated from cholesterol in order to prevent contamination of the endogenous oxysterol content by cholesterol autoxidation products generated during subsequent sample preparation. A Certified Sep-Pak C_18_ column (200 mg) was washed with 4 mL of ethanol then conditioned with 6 mL of 70% ethanol. The brain extract, now in 70% ethanol (3 mL), was applied to the column and allowed to flow at a rate of 0.25 mL/min. Flow was enhanced using a vacuum manifold (Vacuum Processing Station, Agilent, Waghaeusel – Wiesental, Germany). The flow-through was combined with a wash of 4 mL of 70% ethanol giving fraction SPE1-FR1 (7 mL 70% ethanol). Oxysterols elute in this fraction [18,19]. A second wash with another 4 mL of 70% ethanol generated fraction SPE1-FR2. Cholesterol was eluted from the Sep-Pak column with 2 mL of ethanol (SPE1-FR3). A final column stripping with a 2 mL aliquot of ethanol eluted more hydrophobic sterols. (SPE1-FR4). Each fraction from the column (SPE1-FR1→4) was divided into two equal portions giving e.g. SPE1-FR1A, SPE1-FR1B etc. All fractions were dried in a vacuum concentrator and reconstituted in 100 μL of propan-2-ol.

### 2.3 Charge-tagging

**Fig. 1:**
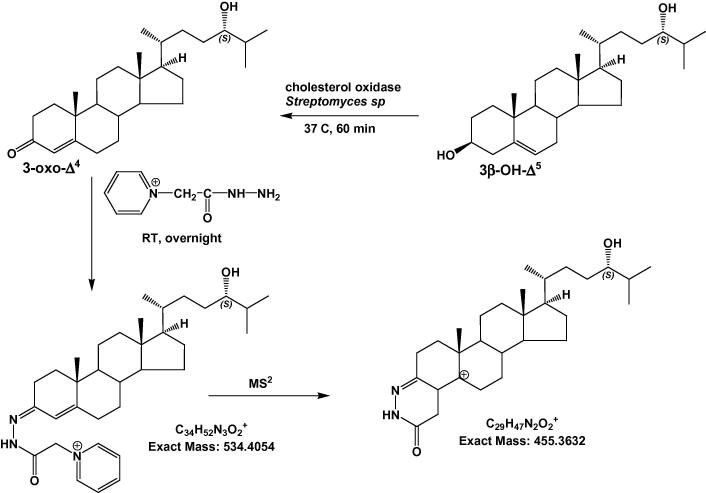
Charge-tagging of oxysterols. The 3β-hydroxy group is oxidised with cholesterol oxidase to a 3-oxo group which is then derivatised with GP hydrazine to give a GP-hydrazone. MS^2^ of the [M]^+^ ion results in a major [M-79]^+^ fragment ion due to loss of the pyridine ring. Further fragmentation of the [M-79]^+^ ion by MS^3^ leads to structurally informative fragment ions, see Supplementary Fig. S1. Oxysterols with a natural 3-oxo group are differentiated from those oxidised to contain one by repeating the charge-tagging procedure in the absence of cholesterol oxidase.

3β-Hydroxy groups in the oxysterols/sterols were first converted to 3-oxo groups using cholesterol oxidase from *Streptomyces* sp*.* (Fig. 1) [20]. A solution of 50 mM phosphate buffer (1 mL, KH_2_PO_4_, pH 7) containing 3.0 μL of cholesterol oxidase (2 mg/mL in H_2_O, 44 units/mg of protein) was added to the A fractions (i.e. SPE1-FR1A→4A). In a similar manner, 50 mM phosphate buffer (1 mL, KH_2_PO_4_, pH 7) but without cholesterol oxidase was added to the B fractions (SPE1-FR1B→4B). The mixtures were incubated at 37 °C for 1 h, and the reaction was terminated with 2 mL of methanol. Oxysterols/sterols possessing an oxo group either naturally or as a result of treatment with cholesterol oxidase were derivatised with GP reagent [18,19]. One hundred and fifty μL of glacial acetic acid and 150 mg of GP hydrazine were added to each of the fractions above, now in 3 mL of ∼70% methanol. The mixture was incubated at room temperature overnight in the dark. The derivatised oxysterols/sterols were separated from excess GP reagent using a recycling method on new C_18_ SPE columns [18,19]. Derivatised oxysterols were ultimately eluted in 2 mL of methanol, while derivatised cholesterol eluted in 3 mL of methanol.

### 2.4 LC–ESI–MS(MS^n^)

The liquid chromatography–electrospray ionisation–mass spectrometry (LC–ESI–MS) system utilised in this study consisted of an LTQ-Orbitrap XL (Thermo Scientific, Hemel Hempstead, UK) equipped with an ESI probe and a Dionex Ultimate 3000 LC system (Dionex, Camberley, Surrey, UK). Chromatographic separation was achieved using a Hypersil Gold RP column (50 × 2.1 mm, 1.9 μm; Thermo Scientific) at room temperature. Injections (5–20 μL) of oxysterols/sterols were made in 60% methanol, 0.1% formic acid. Mobile phase A consisted of 0.1% formic acid in 33.3% methanol, 16.7% acetonitrile. Mobile phase B consisted of 0.1% formic acid in 63.3% methanol 31.7% acetonitrile. Gradient elution was performed as described in [18].

The LC–ESI–MS and LC–ESI–MS^n^ methods were essentially as described earlier [18]. The LTQ-Orbitrap XL was operated utilising three scan events. First a Fourier transform (FT) MS scan was performed in the Orbitrap over the *m*/*z* range 400–605 at 30,000 resolution (full width at half-maximum height definition), in the second event the MS^3^ transition e.g. (534.4→455.4→) was monitored using collision energies of 30 and 35 for MS^2^ and MS^3^, respectively. In the third event another MS^3^ transition e.g. (540.4→461.4→) was monitored in a similar manner (e.g. to accommodate the 24R/S-[^2^H_6_]hydroxycholesterol internal standard). The MS^3^ transitions utilised in the analysis of brain samples are given in Table 1. Oxysterols were quantified with 24R/S-[^2^H_6_]hydroxycholesterol as internal standard, while cholesterol and other hydrophobic sterols were quantified with [^2^H_7_]cholesterol as internal standard. Previous studies by Karu et al. have shown that GP-tagged 3-oxo-4-ene oxysterols (derived by oxidation with cholesterol oxidase from 3β-hydroxy-5-ene precursors) give equivalent response factors in LC–ESI–MS [15].

## 3 Results

### 3.1 WT (Cyp46a1+/+) mice

**Fig. 2:**
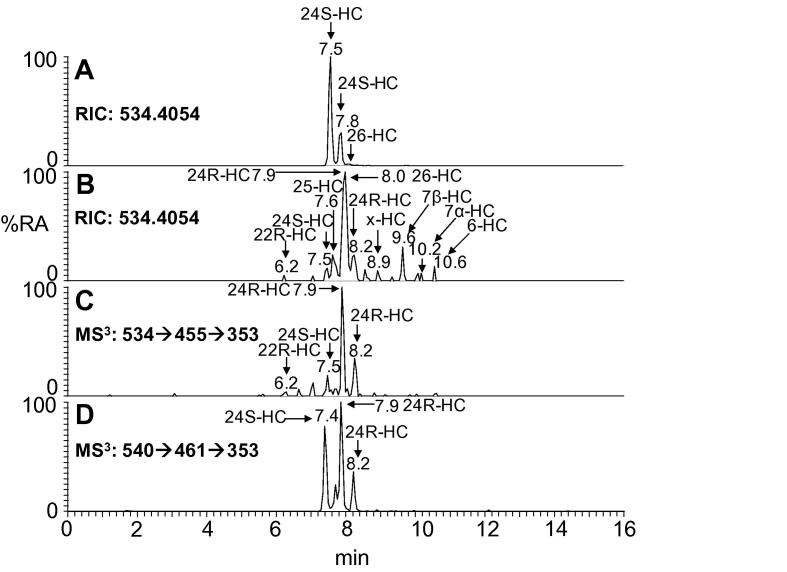
Chromatographic separation of charge-tagged monohydroxycholesterols. RIC for *m*/*z* 534.4054 corresponding to the [M]^+^ ion of monohydroxycholesterols (HC) from (A) *Cyp46a1+*/*+*, and (B) *Cyp46a1*−/−, mouse brain. (C) MRM of the transition 534→455→353 from *Cyp46a1*−/− mouse brain. (D) MRM of the transition 540→461→353 from 24R/S-hydroxycholesterol internal standard.

The reconstructed ion chromatogram (RIC) of *m*/*z* 534.4054 corresponding to the [M]^+^ ion of GP-tagged monohydroxycholesterols from wt mouse brain is shown in Fig. 2A. The chromatogram is completely dominated by GP-tagged 24S-hydroxycholesterol (27.91 ± 0.73 ng/mg, mean ± SD, two mice were analysed in duplicate preparations, Table 1) which, as a consequence of derivatisation, gives two peaks corresponding to the *syn* and *anti* conformers of the GP-derivative. Trace levels of GP-tagged (25R),26-hydroxycholesterol (approx. 0.3 ng/mg) are evident, while low amounts of 25-, 7α- and 7β-hydroxycholesterol also appear in some analysis (⩽0.05 ng/mg). The identification of oxysterols was confirmed by comparison of accurate mass, retention time and MS^n^ spectra with those of authentic standards. As well as the monohydroxycholesterol isomers, 24S,25-epoxycholesterol was also found in brain of the wt animals. During the GP-tagging reactions the 24S,25-epoxide isomerises to the 24-ketone, is hydrolysed to the 24,25-diol and undergoes methanolysis to the hydroxy-methoxide [21]. The level of “total” 24S,25-epoxycholesterol was determined to be 0.64 ± 0.02 ng/mg. Sterols with similar polarity to cholesterol elute from the first SPE column in fraction SPE1-FR3. Analysis of this sterol fraction revealed a high level of cholesterol (16 μg/mg) and a lower level of desmosterol (0.1 μg/mg). Very low levels of the desmosterol precursor 7-dehydrodesmosterol were also observed (0.006 μg/mg).

### 3.2 Cyp46a1−/− mice

**Fig. 3:**
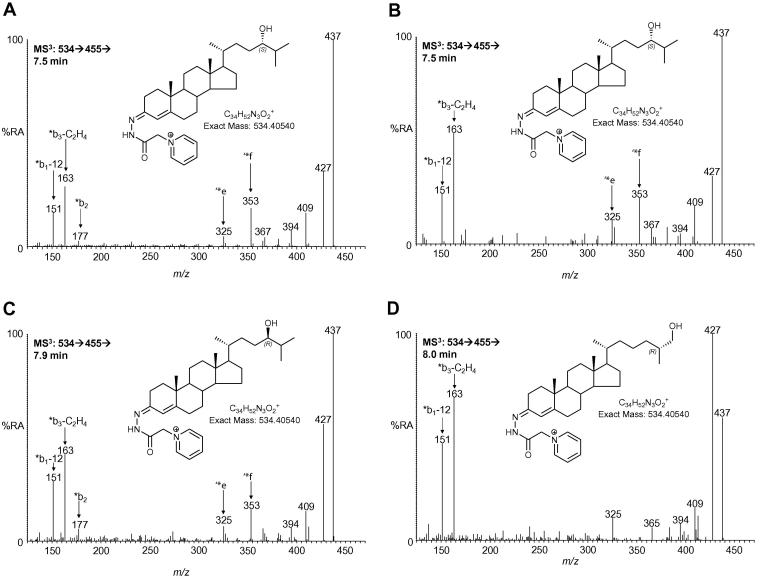
MS^3^ (534→455→) spectra of monohydroxycholesterols in mouse brain. 24S-hydroxycholesterol from (A) *Cyp46a1+*/*+*, and (B) *Cyp46a1*−/− mouse brain. (C) 24R-Hydroxycholesterol and (D) (25R),26-hydroxycholesterol from *Cyp46a1*−/− mouse brain.

In the *Cyp46a1*−/− mouse deletion of the *Cyp46a1* gene results in a great reduction in the level of 24S-hydroxycholesterol in brain (Table 1). This allows the injection of greater amounts of oxysterol “on-column” without the fear of column overloading, effectively increasing the sensitivity of the analytical method from 50 pg/mg to 5 pg/mg. Very low levels of 24S-hydroxycholesterol (0.017 ± 0.003 ng/mg) were observed in brain of the *Cyp46a1*−/− mouse as revealed in the RIC of *m*/*z* 534.4054 corresponding to GP-tagged hydroxycholesterols (Fig. 2B) and by the MS^3^ spectrum shown in Fig. 3B. In the absence of the Cyp46a1 enzyme it may be expected that some other oxysterol would take on the role of the “cholesterol transporter” from brain to liver, however, in agreement with earlier studies [6] there was no matching increases in the abundance of any other oxysterol. As in the wt mouse, low levels of 25- and (25R),26-hydroxycholesterols (0.050 ± 0.009 ng/mL and approx. 0.15 ng/mL, respectively) were evident in the *Cyp46a1*−/− mice (Table 1). Surprisingly, the presence of a metabolite eluting as two components just before, and just after, (25R),26-hydroxycholesterol with retention times and MS^3^ spectra corresponding to 24R-hydroxycholesterol (approx 0.15 ng/mg) was observed (Figs. 2B and 3C). On account of the presence of a fragment ion at *m*/*z* 353 in the MS^3^ spectra of both of the 24-hydroxycholesterol isomers (Fig. 3A–C), but not the 25- or (25R),26-hydroxycholesterol isomers (Fig. 3D), it is possible by generating a multiple reaction monitoring (MRM) chromatogram for the transitions 534→455→353 to “resolve” the 24-hydroxycholesterol isomers from closely eluting 25- and (25R),26-hydroxycholesterols as shown in Fig. 2C (the MS^3^ fragmentation of GP-tagged 24-hydroxycholesterol is shown in Supplementary Fig. S1). The fragment ion of *m*/*z* 353 is also observed in the MS^3^ spectrum of 24R/S-[^2^H_6_]hydroxycholesterol allowing the generation of a MRM chromatogram 540→461→353 (Fig. 2D). There were also low levels of an additional side-chain hydroxylated oxysterol (0.030 ± 0.035 ng/mg) eluting after the second peak of 24R-hydroxycholesterol and before 7β-hydroxycholesterol. The MS^3^ spectrum suggested hydroxylation on a primary or secondary carbon of the side-chain. The final eluting oxysterols from the RIC of 534.4054 are ring hydroxylated and correspond to 7β-, 7α- and 6-hydroxycholesterols. Very low levels of 22R-hydroxycholesterol (<0.005 ng/mg, Fig. 2B), and 20R,22R-dihydroxycholesterol (0.030 ± 0.002 ng/mg) were also evident in the *Cyp46a1*−/− animals. As in the wt animal, 24S,25-epoxycholesterol was found in brain from the *Cyp46a1*−/− animal appearing as the GP-tagged epoxide, its 24-oxo isomer and methanolysis and hydrolysis products (0.124 ± 0.044 ng/mg) .

Despite the absence of appreciable amounts of 24S-hydroxycholesterol, or of a major replacement oxysterol in the brain of *Cyp46a1*−/− mouse, the level of cholesterol was essentially identical to that of the wild-type mouse (16 μg/mg). There was, however, a reduction of desmosterol levels in the *Cyp46a1*−/− mouse (0.06 μg/mg) compared to wt (0.1 μg/mg).

## 4 Discussion

24S-Hydroxylation of cholesterol by Cyp46a1 accounts for about two thirds of cholesterol turnover in mouse brain [22]. Surprisingly, deletion of the *Cyp46a1* gene in the cholesterol 24-hydroxylase knockout mouse does not result in an increase in brain cholesterol but rather a decrease in its rate of synthesis [6]. 24S-Hydroxycholesterol is the dominant oxysterol in wt brain and its high abundance tends to obscure the observation of other oxysterols in brain. The current study, however, was designed to take advantage of a reduction in brain 24S-hydroxycholesterol in the *Cyp46a1*−/− mouse to uncover other oxysterols that may provide an alternative/additional export route for cholesterol from brain or may have regulatory functions in brain.

In brain of the *Cyp46a1*−/− mouse low levels of both 24S- and 24R-hydroxycholesterols were found (Table 1). It is not clear which enzyme(s) generates these isomers and whether their synthesis occurs in brain or if they are imported from the circulation. There are few references to the presence of 24R-hydroxycholesterol in mammals, although Spencer and colleagues have reported that this isomer can be formed enzymatically from 24R,25-epoxycholesterol by Dede cells and by rat liver homogenate [23]. However, 24R,25-epoxycholesterol is not the natural 24,25-epoxide formed via the shunt of the mevalonate pathway in mammals [24]. In the brain of the *Cyp46a1*−/− and also the wt mouse we find low levels of (25R),26-hydroxycholesterol (approx. 0.15 and 0.30 ng/mL, respectively). Cyp27a1, the mitochondrial (25R),26-hydroxylase, is known to be expressed in brain [25]. In brain of wt animals the high level of 24S-hydroxycholesterol (27.91 ± 0.73 ng/mg) and its close chromatographic elution to 25-hydroxycholesterol makes the identification of the latter oxysterol challenging. However, in the absence of the *Cyp46a1* gene the presence of 25-hydroxycholesterol (0.050 ± 0.009 ng/mg) in brain is evident (Fig. 2B). 25-Hydroxycholesterol has recently come to the attention of the lipid community on account of its effects on the immune system [26,27]. Studies show that it is produced and secreted by macrophages in response to TLR activation [26,28]. Migroglia are the resident macrophage of brain and spinal cord and it will be fascinating to see if they show a similar response upon TLR activation.

The enzyme responsible for 7α-hydroxylation of cholesterol, Cyp7a1, is liver specific [2], so the presence of 7α-hydroxycholestertol in brain could be a consequence of diffusion of this oxysterol across the blood brain barrier from the circulation. Both 7α- and 7β-hydroxycholesterols can be formed non-enzymatically from cholesterol [29]. This may be as a result of oxidative stress [30], or alternatively the oxysterols could be autoxidation artefacts formed from cholesterol during sample preparation [31]. Similarly, the 5α and 5β isomers of 5,6-epoxycholesterol are formed from cholesterol non-enzymatically [29]. 5,6-Epoxycholesterols are hydrolysed enzymatically by a microsomal epoxide hydrolase to cholestane-3β,5α,6β-triol. In our charge-tagging procedure 5,6-epoxides undergo hydrolysis and then dehydration and are detected as 6-hydroxycholesterol. In the current study with the *Cyp46a1*−/− mouse we also observe both 22R-hydroxycholesterol and 20R,22R-dihydroxycholesterol in the brain. Cyp11a1 is the enzyme which converts cholesterol into these metabolites.

Oxysterols are in their own right biologically active molecules as ligands to Lxr [11,12], as inhibitors of the processing of SREBPs to their active forms as transcription factors [9], and as modulators of the immune response [26]. Recent studies have shown that Lxr signalling is important for the development of dopaminergic neurons through 24S,25-epoxycholesterol [14]. 24S,25-Epoxycholesterol is present in brain in both the wt and *Cyp46a1*−/− animals, however, the level in the knockout animal is considerably lower than in the wt. This is not surprising as 24S,25-epoxycholesterol is produced in a shunt of the mevalonate pathway by the identical enzymes as cholesterol, and the rate of cholesterol synthesis is reduced in the knockout mouse. Interestingly, symptoms of Parkinson’s disease, the phenotype of dopaminergic neuron loss, have not been observed to-date in the *Cyp46a1*−/− mouse. The reduced level of desmosterol in the brain of the *Cyp46a1*−/− animal is also in agreement with a reduced rate of cholesterol synthesis. Interestingly desmosterol has also been shown to be an Lxr ligand [32,33].

## Figures and Tables

**Table 1:** Oxysterols identified in *Cyp46a1+*/*+ and Cyp46a*−/− mouse brain.

Cmpd	Structure after treatment with cholesterol oxidase	Originating structure systematic name (common name)	[M]^+^ of GP derivative and MS^3^ transition (*m*/*z*)	Rt/min	Cyp46a1+/+ (ng/mg)	Cyp46a1−/− (ng/mg)
1	24S,25-Epoxycholest-4-en-3-one	3β-Hydroxycholest-5-en-24S,25-epoxide (24S,25-epoxycholesterol)	532.3898 532.4→453.3→	6.94	0.03 ± 0.00	0.003 ± 0.001
2	Cholest-4-ene-3,24-dione	3β-Hydroxycholest-5-ene-3,24-dione (24-oxocholesterol)^a^	532.3898 532.4→453.3→	7.92	0.34 ± 0.02	0.005 ± 0.003
3	24,25-Dihydroxycholest-4-en-3-one	Cholest-5-en-3β,24,25-triol (24,25-dihydroxycholesterol)^b^	550.4003 550.4→471.4→	4.09/4.82	0.16 ± 0.00	0.078 ± 0.029
4	24-Hydroxy-25-methoxy-cholest-4-en-3-one	3β,24-Dihydroxycholest-5-ene-25-methoxide^c^	564.4160 564.4→485.4→	6.26/6.74	0.12 ± 0.00	0.037 ± 0.019
**5**	**Sum**	**Total 3β-hydroxycholest-5-en-24S,25-epoxide (24S,25-epoxycholesterol)**^d^			**0.64 ± 0.02**	**0.124 ± 0.044**
6	22R-Hydroxycholest-4-en-3-one	Cholest-5-ene-3β,22R-diol (22R-hydroxycholesterol)	534.4054 534.4→455.4→	6.23	ND	<0.005
7	24S-Hydroxycholest-4-en-3-one	Cholest-5-ene-3β,24S-diol (24S-hydroxycholesterol)	534.4054 534.4→455.4→	7.47/7.78	27.91 ± 0.73	0.017 ± 0.003
8	24R-Hydroxycholest-4-en-3-one	Cholest-5-ene-3β,24R-diol (24R-Hydroxycholesterol)^e^	534.4054 534.4→455.4→	7.89/8.25	Approx ⩽ 0.05	Appox 0.15
9	25-Hydroxycholest-4-en-3-one	Cholest-5-ene-3β,25-diol (25-hydroxycholesterol)	534.4054 534.4→455.4→	7.63	⩽0.05	0.050 ± 0.009
10	26-Hydroxycholest-(25R)-4-en-3-one	Cholest-(25R)-5-ene-3β,26-diol ((25R),26-hydroxycholesterol or 27-hydroxycholesterol)^e^^,^^f^	534.4054 534.4→455.4→	8.04	Approx 0.3	Approx 0.15
11	x-Hydroxycholest-4-en-3-one	Cholest-5-en-3β,x-diol (x-hydroxycholesterol)^g^	534.4054 534.4→455.4→	8.92	⩽0.05	0.030 ± 0.035
12	7β-Hydroxycholest-4-en-3-one	Cholest-5-ene-3β,7β-diol (7β-Hydroxycholesterol)^h^	534.4054 534.4→455.4→	9.64	⩽0.05	0.183 ± 0.243
13	7α-Hydroxycholest-4-en-3-one	Cholest-5-ene-3β,7α-diol (7α-hydroxycholesterol)^i^	534.4054 534.4→455.4→	10.18	⩽0.05	0.040 ± 0.053
14	6-Hydroxycholest-4-en-3-one	Cholest-5-ene-3β,6-diol (6-hydroxycholesterol)^j^	534.4054 534.4→455.4→	10.56	ND	0.053 ± 0.069
15	20R,22R-Dihydroxycholest-4-en-3-one	Cholest-5-en-3β,20R,22R-triol (20R,22R-dihydroxycholesterol)	550.4003 550.4→471.4→	4.51	⩽0.05	0.030 ± 0.002

Oxysterols identified by retention time, exact mass, MS^n^ spectra and comparison to authentic standards. Quantification was made against the internal standard 24R/S-[25,25,25,26,26,26-^2^H_6_]hydroxycholesterol internal standard. Mean concentration ± standard deviation for two Cyp46a1+/+ and two Cyp46a1−/− animals analysed in duplicate.

a Isomerisation product of 24S,25-epoxide during derivatisation.

b Hydrolysis product of 24S,25-epoxide during derivatisation.

c Methanolysis product of 24S,25-epoxide during derivatisation. MS^n^ suggests a 24-hydroxy-25-methoxy structure.

d Total 24S,25-epoxycholesterol, sum of cmpds 1–4.

e Quantification of 24R-hydroxycholesterol and (25R),26-hydroxycholesterol is approximate as the derivatives are only resolved from one another in MRM chromatograms.

f Systematic nomenclature defines the hydroxy group on the terminal carbon of the sterol side chain to be attached to carbon-26 when R stereo chemistry is introduced at carbon-25. However, on account of the enzyme Cyp27 catalysing this hydroxylation, the common name for the hydroxycholesterol is 27-hydroxycholesterol.

g The site of hydroxylation is probably on the side chain at a primary or secondary carbon.

h May be an autoxidation product of cholesterol.

i Can be formed enzymatically or as an autoxidation product of cholesterol.

j Dehydration product of cholest-5-ene-3β,5,6-triol a hydrolysis product of 5,6-epoxycholesterol, an autoxidation product of cholesterol.

## Appendix A Supplementary data

Supplementary data 1 Fragmentation of the [M-79]^+^ ion from 24-hydroxycholesterol isomers.
