# Supplementary figures and images for: Oxysterols in the brain of the cholesterol 24-hydroxylase knockout mouse
Source: Biochem Biophys Res Commun. 2014 Apr 11;446(3):768–74. doi: 10.1016/j.bbrc.2014.01.153 (PMC4000437; doi:10.1016/j.bbrc.2014.01.153)

## Slide 1
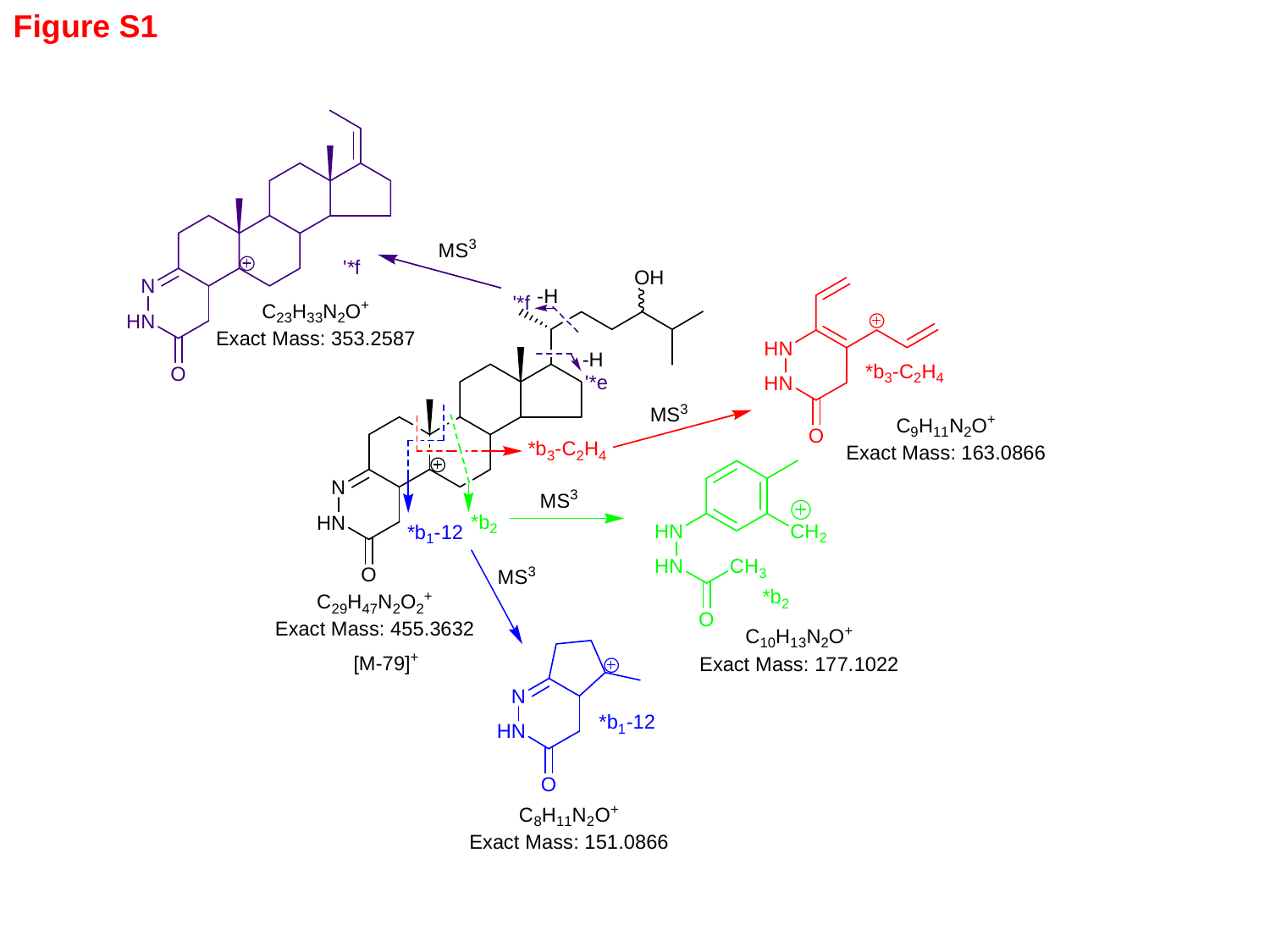

Figure S1

Supplement: Supplementary data 1 — Fragmentation of the [M-79]+ ion from 24-hydroxycholesterol isomers. [file mmc1.pptx]
